# Sexual partnership concurrency and age disparities associated with sexually transmitted infection and risk behavior in rural communities in Kenya and Uganda

**DOI:** 10.1016/j.ijid.2022.04.038

**Published:** 2022-04-25

**Authors:** Jaffer Okiring, Monica Getahun, Sarah A. Gutin, Sarah Lebu, Joi Lee, Irene Maeri, Patrick Eyul, Elizabeth A. Bukusi, Craig R. Cohen, Torsten B. Neilands, Sarah Ssali, Edwin D. Charlebois, Carol S. Camlin

**Affiliations:** 1 Infectious Diseases Research Collaboration, Plot 2C Nakasero Hill Road, P.O. Box 7475, Kampala, Uganda; 2 Department of Obstetrics, Gynecology & Reproductive Sciences, University of California San Francisco, 1330 Broadway, Ste. 1100, Oakland CA 94612, USA; 3 Center for AIDS Prevention Studies (CAPS), Division of Prevention Science, Department of Medicine, University of California, San Francisco, 550 16th Street, 3rd Floor, San Francisco, CA 94158, USA; 4 Centre for Microbiology Research, Kenya Medical Research Institute, P.O. Box 54840 00200, Nairobi, Kenya; 5 School of Women and Gender Studies, Makerere University, Pool Road, Kampala, Uganda

**Keywords:** Age disparity, Sexual risk, STIs, Concurrency, Population mobility, Sub-Saharan Africa

## Abstract

**Objectives:**

We examined sex-specific associations of partner age disparity and relationship concurrency with *Neisseria gonorrhoeae* and/or *Chlamydia trachomatis* (NG/CT) infection, higher-risk relationships, and condom use as proxies for HIV risk.

**Methods:**

Data were collected in 2016 from 2179 adults in 12 communities in Uganda and Kenya. Logistic regression models examined associations of age disparity and relationship concurrency with NG/CT infection, condom use, and higher-risk (commercial sex and other higher-risk) relationships in the past 6 months, controlling for covariates.

**Results:**

Partner age and relationship concurrency were associated with NG/CT infection in women but not men. Relative to women in age-disparate relationships, women in both age-disparate and age-homogeneous relationships had higher odds of NG/CT infection (adjusted odds ratio [aOR]=3.82, 95% confidence interval [CI]: 1.46–9.98). Among men and women, partnership concurrency was associated with higher-risk partnerships. In addition, relative to those with a single age-homogeneous partner, those with concurrent age-homogeneous partners had higher odds of condom use (men: aOR=2.85, 95% CI: 1.89–4.31; women: aOR=2.99, 95% CI: 1.52–5.89). Concurrent age-disparate partnerships were associated with condom use among men only (aOR=4.02, 95% CI: 2.54–6.37).

**Conclusion:**

Findings underscore the importance of targeted HIV prevention efforts for couples in age-disparate and concurrent relationships.

## Background

While global trends in HIV infection point to declining HIV incidence in many world regions, incident HIV infection persists in sub-Saharan Africa. A disproportionate burden of HIV among key populations exists, including among women and girls, who account for 59% of all new HIV infections and a high proportion of people living with HIV in Eastern and Southern Africa (12.3 million women aged ≥15 years compared with 7.3 million men aged ≥15 years) (Karim and Baxter, 2019; Scully, 2018; Sia et al., 2020; UNAIDS, 2020). Sex-specific factors for this disparate outcome include not only biological (Liebenberg et al., 2019) but social-behavioral drivers for young women, including early age of sexual debut, unprotected sex, and limited agency and relational decision-making (Kidman and Kohler, 2019; Maughan-Brown et al., 2019; Mwinnyaa et al., 2018). Furthermore, sexually transmitted infections (STIs), which often have a higher prevalence in women than in men (Buvé et al., 2007), have been shown to increase HIV acquisition risk and transmission (Dubbink et al., 2018; Jongen et al., 2021; Kharsany et al., 2020) and can serve as a biomarker for unprotected sexual intercourse (Osinde et al., 2012).

Equally important and less researched is the role of age-disparate relationships—having at least a five-year gap in the age of partners—in HIV acquisition and transmission (Leclerc-Madlala, 2008; UNAIDS, 2015). Although HIV incidence among young women is partly attributed to being in sexual relationships with older men (Maughan-Brown et al., 2019; Mwinnyaa et al., 2018), research examining the interplay between age disparity and HIV incidence has produced mixed results. Studies suggest that be-fore widespread antiretroviral therapy use, young women in age-disparate relationships were at elevated risk of HIV infection because of their older partners’ burden of HIV, STIs, and the occurrence of riskier sexual behavior such as transactional and condomless sex and concurrent relationships (Evan et al., 2016; Maughan-Brown et al., 2016; McKinnon and Karim, 2016; Mwinnyaa et al., 2018). In these relationships, younger women often lack the agency to negotiate safer sex because of power differentials and the transactional nature that define them (Maughan-Brown et al., 2014). Conversely, for young women, associations have been reported between age-disparate relationships and a decreased risk of HIV acquisition from people living with HIV (Maughan-Brown et al., 2018; Street et al., 2016). This research posits that HIV testing is positively correlated with age, and therefore, in the context of U=U (Undetectable = Untransmittable/uninfectious), older men are more likely to be diagnosed, linked to HIV care, and virally suppressed, and unlikely to pose additional risk of infection for young women in comparison with their younger counterparts (Akinyi et al., 2017; Govender et al., 2019; Houle et al., 2018; Maughan-Brown et al., 2018; Youssef et al., 2018). A study in KwaZulu-Natal found that age-disparate relationships protected young women from increased risk of HIV acquisition (Harling et al., 2014). Thus, whether male partners’ older age is protective or presents potentially higher HIV risk to younger women may depend on levels of HIV care engagement and viral suppression among men living with HIV in a population.

Furthermore, relationship concurrency—any temporal overlap of one or more sexual relationships—has the potential to compound HIV risk, especially among women in age-disparate relationships where their partners have multiple other partners (Steffenson et al., 2011; Tanser et al., 2011; N and A, 2015). The number of partners in a network increases the cumulative odds of one or more partners having HIV and a detectable viral load, increasing the risk of HIV acquisition (McKinnon and Karim, 2016). However, if older male partners are virally suppressed and connected to care, they may not spread HIV within concurrent relationships. Although there may be synergistic effects between age-disparate relationships and concurrency that may increase the scale of the HIV epidemic, the literature is inconclusive.

The complex dynamic of age disparity and sexual and behavioral risks, further complicated by the dynamics of mobility, which shapes and interacts with the sexual behaviors of women and men, geographically links centers of infection, incentivizes riskier sexual behavior, and disrupts ongoing HIV care (Camlin et al., 2019; Camlin and Charlebois, 2019; Deane et al., 2010; Isdory et al., 2015; Kreniske et al., 2019; Schuyler et al., 2017; Vissers et al., 2008). Mobility can therefore negate some of the benefits that might otherwise be achieved from HIV care engagement.

Therefore, among a highly mobile population in Uganda and Kenya, we sought to measure associations of partner age-mixing (i.e., sex with people outside of one’s age group) and partnership concurrency with the outcome of higher-risk sexual behaviors, using three indicators as proxies for HIV exposure risk: (1) active *Chlamydia trachomatis* (CT) and/or *Neisseria gonorrhoeae* (NG) infection and (2) higher-risk partnerships (defined as relationships reported as casual sex, one-night stands, commercial sex worker/client, or inherited partner), and (3) condom use. In this way, we hoped to shed light on whether age-disparate relationships are protective for women because their older partners are virally suppressed or whether age-disparate relationships and concurrency are continuing to place women at increased HIV risk.

## Methods

### Study design and setting

This study leveraged the Sustainable East Africa Research in Community Health (SEARCH) trial (NCT# 01864603), a six-year cluster-randomized trial in 32 communities in three regions in Kenya and Uganda, to evaluate the effectiveness of a universal testing and treatment approach for reducing community HIV incidence (Havlir et al., 2019). This study was embedded within 12 of the 32 SEARCH communities and measured the mobility of individuals in these communities and the impact on HIV incidence and the HIV care cascade.

A multi-level stratified random sampling design based on SEARCH study arm (intervention or control), HIV status (positive or negative), and mobility (non-mobile or mobile) was used to select the sample from the adult population of each of the 12 SEARCH communities. The 12 communities were selected purposively to reflect underlying heterogeneity in forms of mobility across SEARCH communities and were composed of three communities each from two regions of Uganda and three inland and three Lake Victoria shoreline communities in Kenya. Mobile and HIV-positive individuals were oversampled to achieve the desired sample size in each stratum.

Ethical approvals were granted by the Ethical Review Committee of the Kenya Medical Research Institute (KEMRI/SERU/CMR/3052), Makerere University School of Medicine Research and Ethics Committee (2015-040), the Uganda National Council for Science and Technology (HS 1834), and the University of California San Francisco Committee on Human Research (14-15058). All participants provided written informed consent to participate in this study.

### Eligibility criteria

Study inclusion was restricted to individuals in the 12 selected SEARCH communities aged ≥16 years, for whom baseline HIV serostatus and mobility status were available. HIV status was ascertained using rapid, finger-prick blood-based HIV antibody testing and counseling following Ministry of Health of Uganda guidelines.

### Data collection

Mobility and sexual risk behavior survey data as well as urine samples to screen for CT and NG were collected during a baseline visit between February and November 2016. Of 2750 possible study participants, 2601 agreed to provide urine samples. Data were collected within rural communities with varying levels of geographic mobility and at the participant’s preferred location; research assistants visited participants to collect survey data and biological specimens. Survey data were collected using custom-designed Microsoft Windows forms and an Access database on programmed tablets, and took about one and a half hours to complete. The study used a novel survey instrument, described in detail elsewhere (Camlin et al., 2018; Camlin et al., 2019), that was developed to measure the complex forms of movement that are emergent in low- and middle-income country settings such as Eastern Africa, including women’s mobility. Participants were asked about their histories of migration over their lifetimes and mobility in the past 6 months by purpose, location, and frequency. The study also developed a Relationship History Calendar survey (Camlin et al., 2018), adapted from an instrument previously used in the region and shown to reduce social desirability bias to improve the reporting of sexual relationships and behavior (Luke et al., 2011), to collect information about sexual behavior and partnerships in the past 5 years; data were collected month by month on the type of relationships and sexual behaviors with each partner, including condom use. Before data collection, the customized electronic questionnaire was piloted and pre-tested. For participants who provided urine samples, 7 ml of urine were pipetted and transferred into manufacturer-provided transport reagent tubes containing a buffer solution/preservative and tightened securely. Urine samples were stored in refrigerated (at 2-8°C) boxes while in the field and transported to a regional laboratory (Mbita in Kenya, Mbarara in Uganda) twice a week. Samples were kept in a laboratory fridge at 2-8°C for up to 45 days in accordance with guidelines on manufacturer-provided urine transport tube. Most samples were processed within one to 2 weeks of receipt at the laboratory and screened for CT/NG using the GeneXpert^®^ CT/NG reverse transcription-polymerase chain reaction assay (Cepheid, Sunnyvale, CA) (Tabrizi et al., 2013).

### Data analysis

#### Independent variables

The primary independent exposure variables of interest were (1) age-disparate relationships in the past 6 months, as defined by the Joint United Nations Programme on HIV/AIDS as relationships with a ≥5-year age disparity between the partners (UNAIDS, 2015) and (2) partnership concurrency in the past 6 months. We generated seven different variables, which are described in Table 1.

#### Dependent variables

Three outcome measures of sexual risk behaviors were assessed: (1) active CT and/or NG infection at the time of sample collection, (2) higher-risk sexual relationship (defined as relationships that were reported as casual sex, one-night stands, commercial sex worker/client, or inherited partner, excluding concurrent partnerships) in the past 6 months, and (3) any condom use in the past 6 months.

Statistical analyses were performed using Stata version 16.1 (StataCorp, College Station, TX, USA). Characteristics of the study populations were summarized by sex. Bivariate comparisons that accounted for clustering of individuals within communities (Rao-Scott F-tests) were used to characterize the relationship between characteristics of interest and sex. Sex-stratified logistic regression models with robust standard errors were used to ascertain associations between age-disparity classifications and sexual risk, controlling for age, occupation, household wealth index, HIV status, and mobility in the past 6 months and adjusting for community clustering. All variables that were significant at the bivariate level and the main exposures were included in model building. The best model was selected on the basis of the Akaike information criterion (because it is less likely to miss potentially important variables). Measures of association are expressed as adjusted odds ratios (aORs) and *P*-values (two-sided) of <0.05 considered statistically significant. All observations were used in models, but some models excluded observations that predicted the outcome perfectly and thus had smaller sample sizes. For the sensitivity analysis, because of the small numbers of positive responses for some variables in models involving the higher-risk sexual relationship variable, we refitted penalized maximum likelihood estimation methods (Heinze, 2006). Confidence intervals (CIs) were generated via cluster bootstrapping based on 5000 bootstrap samples.

## Results

### Demographic and relationship characteristics

#### Demographics

Of the 2750 participants enrolled in the study, 2179 (79.2%) had complete survey and relationship calendar history data, including partner age, and had a relationship within the past 6 months, and were thus included in our analyses. In addition, of those who had complete relationship calendar history data, 2082 had provided a urine sample to screen for CT/NG. Women comprised 51.2% of the sample. The age composition of the sample reflected the underlying population structure, with somewhat higher proportions of women (34.5%) than of men (24.6%) aged 25–34 years and slightly higher proportions of men (28.5%) than of women (26.3%) aged 35–44 years. A large proportion of participants (81.3%) were exclusively in occupations of relatively low HIV acquisition risk such as farming and shop keeping, whereas 18.7% were in informal occupations of higher HIV acquisition risk such as trucking and fishing. Most men and women (76.7% and 86.1%, respectively) were engaged in lower-risk employment compared with those in informal higher-risk employment (23.3% and 13.9% respectively, *P* < 0.001). Most women (81.2%) and men (72.1%) completed primary level education, whereas 18.8% of women and 27.9% of men completed a secondary-level education. Some men (15.3%) and women (14.0%) had household wealth indices in the lowest quintile (Table 2).

#### Mobility and travel

Overall, 53.9% reported travel in the past 6 months (Table 2). Overall, more women (56.6%) than men (33.7%) traveled for non–work-related reasons, whereas more men (23.1%) than women (2.7%) traveled for work-related reasons. There was a significant difference in travel in the past 6 months by sex, with more women (58.0%) than men (49.9%) reporting travel for either work or non–work-related reasons (*P*<0.001) (Table 2).

#### Distribution of age-disparate relationships

**Figure 1.**
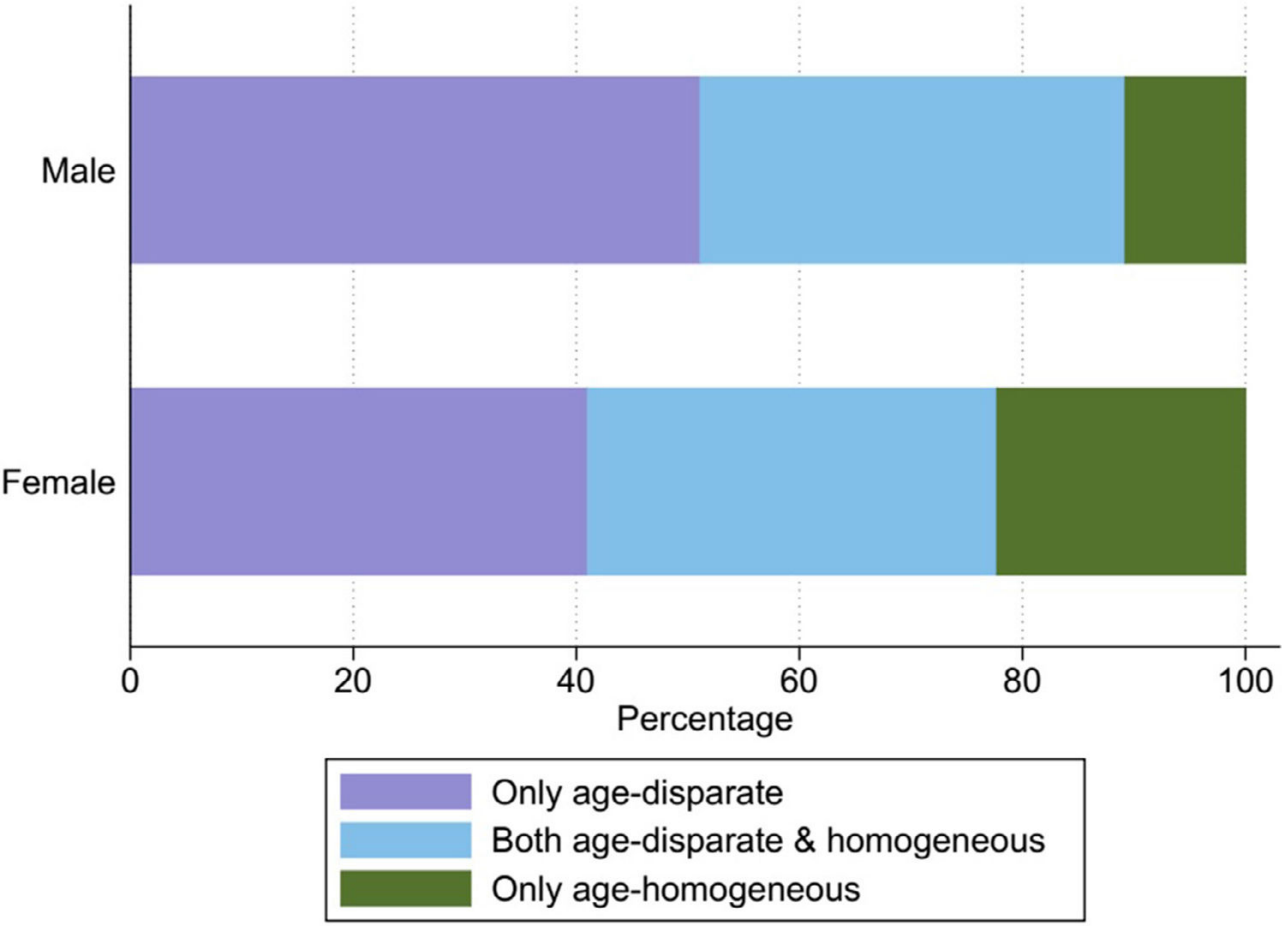
The distribution of partner age-difference classifications by sex; x-axis (proportions), y-axis (sex).

Overall, 57.6% of participants reported one sexual relationship, while 21.6% reported having more than one sexual relationship in the past 6 months (data not shown). Most relationships were only age-disparate (46.2%), compared with both age-disparate and homogeneous (37.4%), and only age-homogeneous (16.4%). There were significant sex differences in the patterns of relationship age disparity reported in the last 6 months. More men (51.1%) than women (41.0%) reported age-disparate relationships (*P*<0.001) (Figure 1). Among women, a greater proportion reported age-disparate (41.0%) than both age-disparate and homogeneous (36.7%) or age-homogeneous (22.3%) relationships (*P*<0.001). Reflecting the age-sex mixing pattern in the population, a similar pattern was observed among men, with a greater proportion reporting age-disparate relationship compared with those reporting both age-disparate and homogeneous and age-homogeneous relationships (all *P*<0.001) (Figure 1).

#### Distribution of concurrent sexual relationships

**Figure 2.**
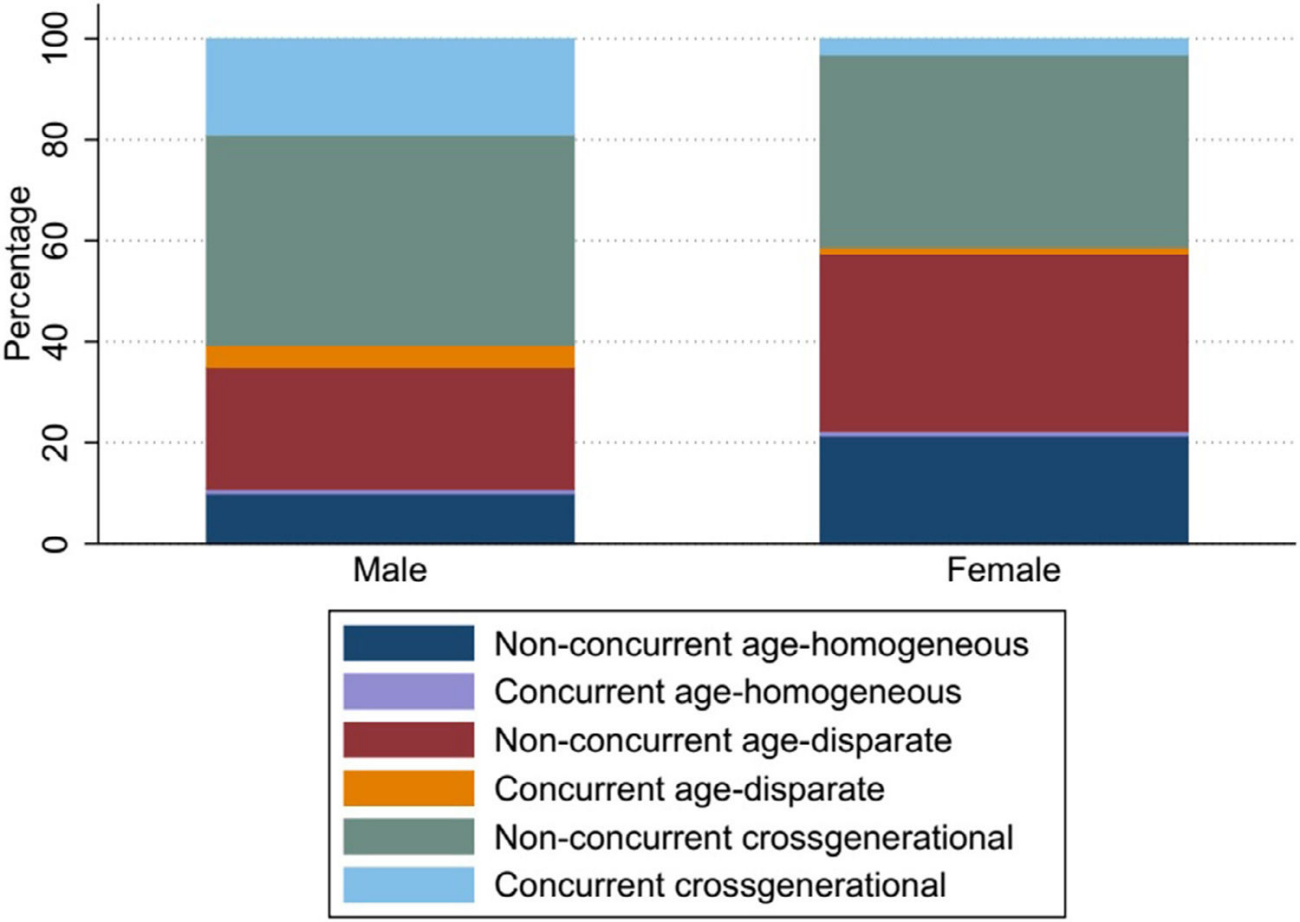
The distribution of partner age-difference classifications with relationship concurrency by sex; x-axis (sex), y-axis (proportions).

Among the participants with sexual relationships in the past 6 months, 15.1% had at least one concurrent relationship; more men (24.2%) than women (5.6%) reported concurrency (*P*=0.001). Figure 2 shows the distribution of age-difference classifications with concurrency by sex. As shown, larger proportions of men than women reported concurrent partnerships of all types in the past 6 months. Specifically, 23.4% of men vs 4.5% of women reported concurrent age-disparate partners, 14.8% of men vs 4.3% of women age-homogeneous concurrent partners, and 19.0% of men vs 3.2% of women concurrent cross-generational partners (all *P*<0.001) (Table 2).

### Higher-risk sexual partnerships, CT/NG, and condom use

Overall, 3.4% of the participants had a positive test for CT (51.4%), NG (44.3%), or both (4.3%) (Table 3). Almost 10% reported having at least one higher-risk relationship in the past 6 months (casual sex partner, one-night stand, commercial sex, or other higher-risk partner), with more men (24.3%) than women (5.5%) reporting these types of partners (*P*<0.001). Overall, 32.5% of participants reported using a condom in the past 6 months, with more men (27.5%) than women (19.7%) reporting condom use (*P*<0.001).

### Effect of age disparity on sexual risk behaviors by sex

Table 4 shows results of sex-specific multiple logistic regression models examining associations between age-disparate relationship classifications and outcomes of CT/NG (5 models), higher-risk sexual partnerships (5 models), and condom use in past 6 months (5 models), respectively. As shown, there were pronounced independent associations of partner age and partnership concurrency with active CT/NG infection in women, but not in men. Relative to women in age-disparate relationships, women in age-homogeneous relationships had nearly three-fold higher odds of active CT/NG infection (aOR=2.87, 95% CI: 1.18–6.98), and women reporting both age-disparate and age-homogeneous relationships had almost four-fold higher odds of CT/NG infection (aOR=3.82, 95% CI: 1.46–9.98). Independently, relative to women with only one age-homogeneous relationship in the past 6 months, women in concurrent cross-generational relationships had more than six-fold higher odds of CT/NG infection (aOR=6.18, 95% CI: 1.54–24.83) (Table 4). Figure 3a presents the effect sizes of mutually classified variables of concurrent age disparity in CT/NG occurrence by sex. The observed effect sizes varied by sex, with greater effects among women than among men.

In contrast, among both men and women, sexual partnership concurrency was very highly associated with having also had a higher-risk sex partner in the past 6 months. Relative to those in nonconcurrent age-homogeneous relationships, there were significant associations between concurrent age-disparate partner-ships and higher-risk partnerships in men (aOR=13.02, 95% CI: 5.06–33.46) and between concurrent cross-generational relationships and higher-risk partnerships in both men (aOR=17.77, 95% CI: 7.00–45.19) and women (aOR=16.55, 95% CI: 5.99–45.70) (Table 4). Relative to their counterparts with a single age-homogeneous partner in the past 6 months, those with concurrent partners of all types had higher odds of at least one of those partners having been a higher-risk partner. Sensitivity analysis results (data not shown) aligned closely with the main results presented previously and yielded identical substantive conclusions, lending additional support to the main results shown in Table 4. Figure 3b presents the effect size of mutually classified variables of concurrent age disparity in higher-risk partnerships by sex. The observed effect sizes are fairly evenly distributed by sex, with the exception of age-homogeneous relationships where a greater effect was observed in men than in women.

Similarly, relative to women in age-disparate relationships, women in age-homogeneous relationships had higher odds of reporting any condom use in the past 6 months (aOR=1.55, 95% CI: 1.03–2.33). Associations between concurrent age-disparate partnerships and any condom use in the past 6 months were only significant among men (aOR=4.02, 95% CI: 2.54–6.37). Relative to their counterparts with a single age-homogeneous partner in the past 6 months, those with concurrent age-homogeneous partners had higher odds of reporting condom use among both men (aOR=2.85, 95% CI: 1.89–4.31) and women (aOR=2.99, 95% CI: 1.52–5.89). A similar pattern was observed among those with a single cross-generational partner in the past 6 months, and those with concurrent partners of all types had higher odds of reporting condom use among both men and women (Table 4).

## Discussion

This study reveals how age-mixing patterns and concurrency in sexual partnerships are associated with higher-risk sexual behaviors (such as CT/NG infection, having a higher-risk sexual partner, and lack of condom use) that can be used as proxies for HIV acquisition risk among adults in rural Eastern African communities. Importantly, these associations differed by sex and reveal highly gendered patterns of partner selection and sexual behavior.

Men were much more likely than women to report concurrent partnerships, whereas women were more likely to report just one partner in the past 6 months. Larger proportions of men than women were in both nonconcurrent and concurrent cross-generational partnerships (i.e., most often, the male partner was ≥10 years older than female partner), whereas larger proportions of women than men were in nonconcurrent partnerships, including both age-homogeneous and age-disparate partners (most often, male partner ≥5 years older than female partner). Very small proportions of both men and women had concurrent partners who were age-mates.

In this study, women in relationships with both older and same-age partners had significantly greater odds of having active CT/NG infection in the past 6 months—a finding consistent with prior South African research (Beauclair et al., 2012; Street et al., 2016). In addition, women had greater odds of having active CT/NG infection if they were in a concurrent age-homogeneous, age-disparate, or cross-generational relationship, suggesting that in this setting, irrespective of age difference, the risk of STIs as a result of any relationship concurrency is high and may increase the risk of HIV acquisition or onward transmission. Prior research has shown individual-level associations between concurrent relationships and HIV infection risk (Kenyon et al., 2016) due to the heightened risk of potential exposure via several partners rather than differences in specific sexual behaviors (Stoner et al., 2019). Although almost all types of age-disparate or concurrent relationships increase the risk for active CT/NG infection among women, the same effect was not present for men, irrespective of the type of relationship, age disparity, or concurrency. Young women in particular may be more susceptible to STIs for a host of biological and sociological reasons (Buvé et al., 2007), whereas men may be less susceptible for various reasons (e.g., less efficient female-to-male transmission) (Buvé et al., 2007). Although it is hypothesized that older men who are virologically suppressed might pose less HIV acquisition risk to younger women, CT/NG results as a proxy for unprotected sex can indicate the occurrence of condomless sex. Therefore, although women’s risk of HIV acquisition may depend on the level of viral suppression in their partners, they remain at risk of unintended pregnancies and STIs and their associated sequela.

Those with concurrent and age-disparate relationships were also more likely to report having higher-risk partners (such as casual or commercial sex partners), thus increasing their potential exposure to HIV infection or onward transmission of HIV. Although this study did not explore associations with HIV infection directly, evidence suggests that age-disparate relationships may increase HIV infection risk, particularly for young women (Bajunirwe et al., 2020; Maughan-Brown et al., 2019, 2018; Mwinnyaa et al., 2018). Both women and men had greater odds of having a higher-risk partner regardless of whether their concurrent relationships were age-homogeneous, age-disparate, or cross-generational. However, the odds of having a higher-risk partner were almost always higher for men. It is possible that these risks are linked to population mobility. We have previously reported high levels of population migration and shorter-term mobility in this population (with more work-related travel conducted by men and more non–work-related travel reported by women) and highly gendered associations of higher-risk sexual behaviors with mobility (Camlin et al., 2018). In particular, we have reported high levels of relationship concurrency in this population, with men reporting more overall concurrency than women (Camlin et al., 2018). It is possible that high levels of population mobility are contributing to men and women having greater opportunities for concurrent and higher-risk partnerships.

Our findings on condom use also point to the gendered nature of age-disparate relationships. In this study, women had increased odds of using a condom in an age-homogeneous relationship, and both men and women had approximately equal odds of condom use within concurrent age-homogeneous relationships, suggesting, as might be expected, that women have more agency in condom use in relationships with age-mates. However, men had greater odds of condom use in age-disparate relationships, while there was no significant effect for women, suggesting that men have more control over condom use in these relationships, whereas women may not be able to negotiate safer sex because of power differentials (Maughan-Brown et al., 2014). Interestingly, both men and women had approximately equal odds of using condoms in cross-generational relationships. These relationships might be assumed as the least equal in terms of social power or as the most transactional in nature, but both men and women might be aware of the higher risk of STIs, HIV, and unintended pregnancy in these relationships and therefore use condoms for protection.

Our findings, particularly those on CT/NG infection and higher-risk partnerships, support the notion that age-disparate relationships involve unprotected sex, which may fuel HIV transmission. Although our findings present evidence of an association between age-disparate relationships, concurrent relationships, and higher-risk sexual behavior, the precise mechanisms that underlie potential causation have not been definitively explored in this study.

This study had many strengths but also some limitations. First, there was potential of misreporting relationship types and possible error in the reporting of partners’ age. Data were collected from the index participant, suggesting the information provided may have been susceptible to recall bias. To minimize such bias, we only ascertained participants’ partner histories for the past 6 months. In addition, although HIV incidence is an essential outcome of clinical importance, this study in a sample of residents in 12 communities was not powered to detect differences in HIV incidence; the larger intervention trial in 32 communities in which this study was embedded found that cumulative HIV incidence was low and declined by 32% over a three-year period to 0.77% in the intervention and 0.81% in the control groups (Havlir et al., 2019). Finally, the sampling design of this study reflected its purpose of ascertaining the relationship of high-resolution measures of mobility with outcomes of interest, including measures of sexual risk behavior; the study did not aim to develop generalizable population estimates of levels of mobility or of risk behavior.

## Conclusion

Relationship age disparity and relationship concurrency have important gendered associations with NG/CT infection, higher-risk sexual partnerships, and condom use. These findings have policy implications and highlight the relevance of gender-targeted and inclusive interventions to reduce sexual risk behaviors among communities in East Africa. These interventions should also be multi-dimensional given the complex collection of motives that prompt men and women to engage in age-disparate relationships (Leclerc-Madlala, 2008). Our findings underscore the importance of targeted HIV prevention efforts for couples in age-disparate and concurrent relationships, with a focus on mobile populations being especially warranted.

## Figures and Tables

**Figure 3a.**
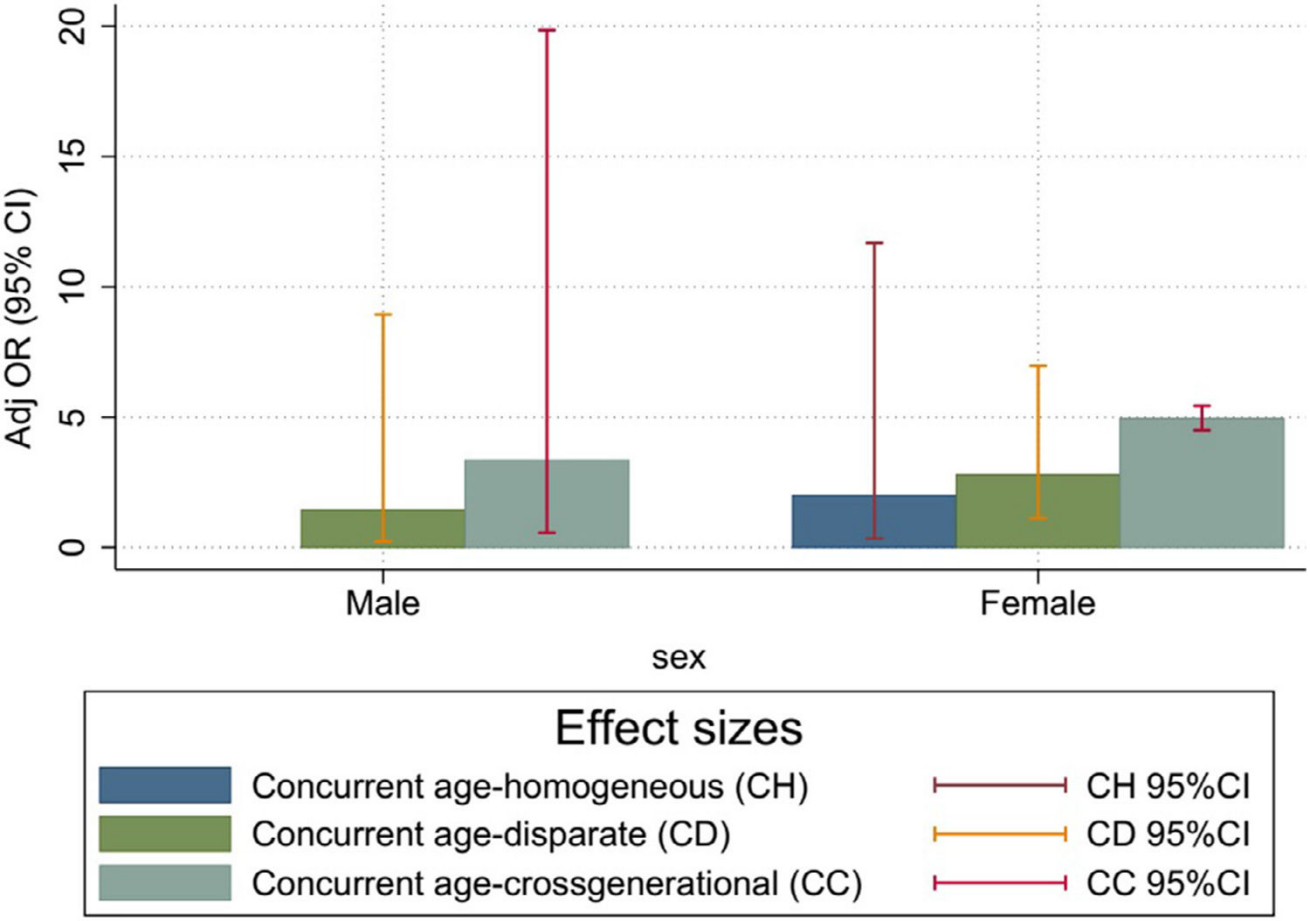
Effect size of mutually classified variables of concurrent and age-disparate relationships in STI occurrence by sex. STI, sexually transmitted infection.

**Figure 3b.**
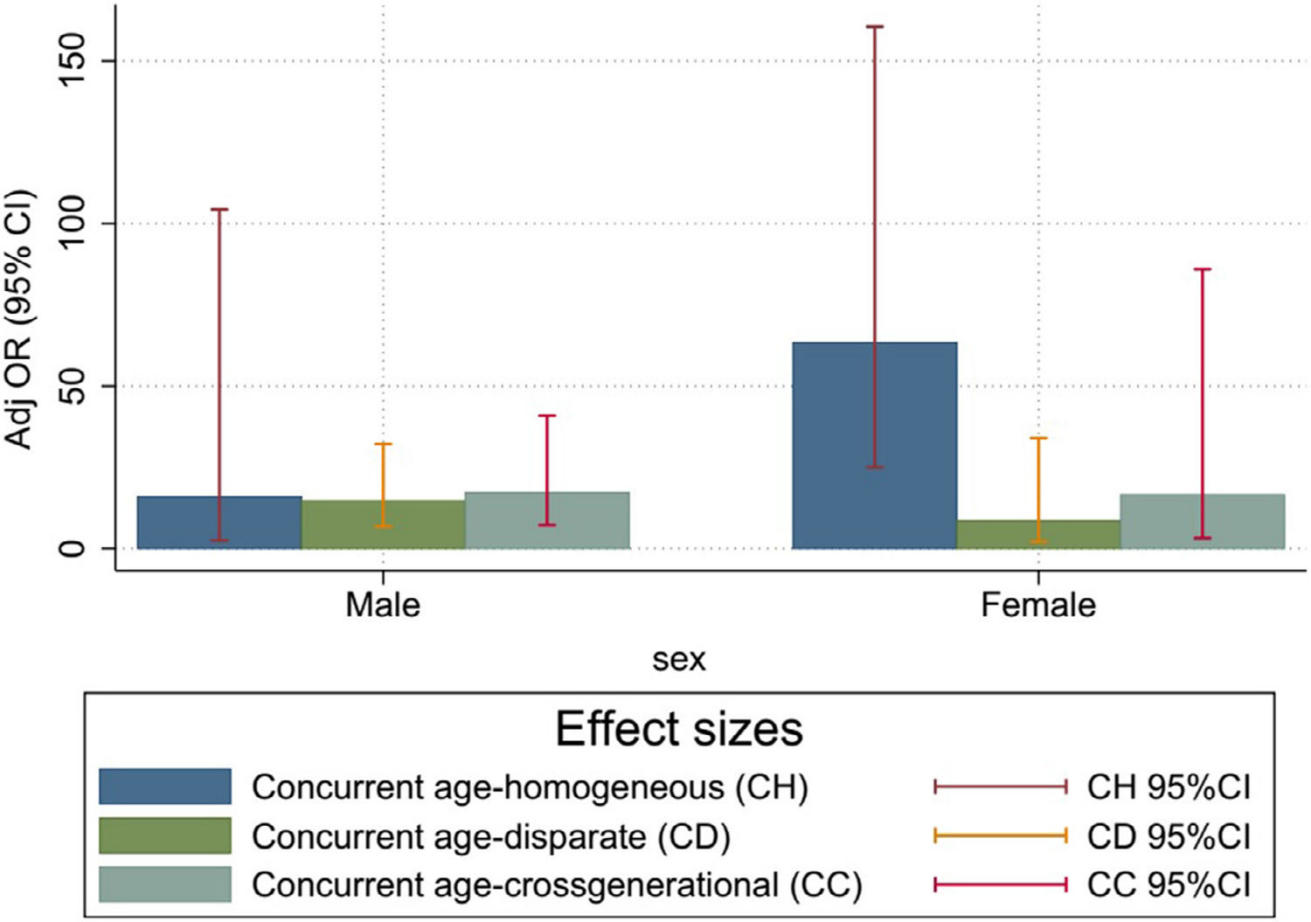
Effect size of mutually classified variables of concurrent and age-disparate relationships in high-risk relationships by sex.

**Table 1:** Independent variables of interest looking at age disparity in relationships.

	Variable	Variable definition	Variable levels
1	Any age-homogeneous relationships	Defined as those with an age difference of <5 years	Any/none
2	Any age-disparate relationships	Defined as those with an age difference of >5 years	Any/none
3	Age disparity with three levels		Age-homogeneous, only age-disparate, and both age-disparate and homogeneous
4	Any concurrent age-homogeneous relationship	Defined as those with a concurrent relationship with an age difference of <5 years	Any/none
5	Any concurrent age-disparate relationship	Defined as those with a concurrent relationship with an age difference of >5 years	Any/none
6	Any concurrent cross-generational relationship	Defined as those with a current relationship with an age difference of > 10 years	Any/none
7	Mutually exclusive variable composed of six levels		Nonconcurrent age-homogeneous, concurrent age-homogeneous, concurrent age-disparate, nonconcurrent age-disparate, concurrent cross-generational, and nonconcurrent cross-generational

**Table 2:** Demographic, mobility, and sexual behavior characteristics of index participants with at least one relationship in the past 6 months, by sex and adjusted for community clustering.

Characteristic	Category	Total, n (%)	Male, n (%) (N=1116)	Female, n (%) (N=1063)	P-value
Demographics					
Age group	15–24	277 (12.7)	101 (9.1)	176 (16.6)	*<0.001*
	25–34	642 (29.5)	275 (24.6)	367 (34.5)	
	35–44	597 (27.4)	318 (28.5)	279 (26.3)	
	45–54	360 (16.5)	212 (19.0)	148 (13.9)	
	≥55	303 (13.9)	210 (18.8)	93 (8.7)	
Employment risk level	Low risk	1771 (81.3)	856 (76.7)	915 (86.1)	*<0.001*
	Informal higher risk	408 (18.7)	260 (23.3)	148 (13.9)	
Educational attainment	At most primary	1668 (76.6)	805 (72.1)	863 (81.2)	*0.001*
	Secondary and above	511 (23.4)	311 (27.9)	200 (18.8)	
Wealth Index	All other wealth quintiles	1859 (85.3)	945 (84.7)	914 (86.0)	0.389
	Poorest wealth quintile	320 (14.7)	171 (15.3)	149 (14.0)	
Regional distribution	Kenya	1222 (56.1)	629 (56.4)	593 (55.8)	0.581
	Uganda East	399 (18.3)	288 (25.8)	270 (25.4)	
	Uganda South-west	558 (25.6)	199 (17.8)	200 (18.8)	
HIV status	Negative	1309 (60.1)	650 (58.2)	659 (62.0)	0.008
	Positive	870 (39.9)	466 (41.8)	404 (38.0)	
Polygamy status	Yes	383 (17.6)	175 (15.7)	208 (19.6)	0.017
	**Age disparity of participants**				
Any age-disparate relationship (≥5 years)	Yes	1821 (83.6)	995 (89.2)	826 (77.7)	*<0.001*
Any cross-generational relationship (≥10 years)	Yes	1116 (51.2)	677 (60.7)	439 (41.3)	*<0.001*
Age disparity of reported relationships	Age-disparate (≥5 years)	1006 (46.2)	570 (51.1)	436 (41.0)	*<0.001*
	Both homogeneous and disparate	815 (37.4)	425 (38.1)	390 (36.7)	
	Age-homogeneous (0–4 years)	358 (16.4)	121 (10.8)	237 (22.3)	
Concurrent age-disparate relationships in the past 6 months	Yes	309 (14.2)	261 (23.4)	48 (4.5)	*<0.001*
Concurrent age-homogeneous relationships in the past 6 months	Yes	211 (9.7)	165 (14.8)	46 (4.3)	*<0.001*
Concurrent cross-generational relationships in the past 6 months	Yes	246 (11.3)	212 (19.0)	34 (3.2)	*<0.001*
	**Mobility of participants**				
Any work or nonwork travel in the past 6 months	Yes	1174 (53.9)	557 (49.9)	617 (58.0)	*<0.001*
Any work-related travel in the past 6 months	Yes	287 (13.2)	258 (23.1)	29 (2.7)	*<0.001*
Any non-work-related travel in the past 6 months	Yes	978 (44.9)	376 (33.7)	602 (56.6)	<0.001
Travel type in the past 6 months	No travel	1005 (46.1)	559 (50.1)	446 (42.0)	<0.001
	Work-related	196 (9.0)	181 (16.2)	15 (1.4)	
	Non–work-related	887 (40.7)	299 (26.8)	588 (55.3)	
	Both work and non-work-related	912 (4.2)	77 (6.9)	14 (1.3)	

Age-homogeneous=difference of 0–4 years in age; age-disparate=difference of >5 years in age; cross-generational=difference of >10 years in age. Work-related travel=for earning money or looking for work; non–work-related travel=for any other reason such as caregiving, attending funeral, and social functions. Among the men in polygamous marriages (15.7%), 68.6% (120/175) reported a concurrent relationship, whereas among the women, 5.3% (11/208) reported a concurrent relationship (defined as having had two or more sexual partners in the preceding 6 months).

**Table 3:** Sexual risk behaviors and STI among index participants by sex and adjusted for community clustering.

Characteristic	Category	Total (%)	Male (%) (N=1116)	Female (%) (N=1063)	*P*-value
Active STI (CT/NG)^a^ (n=2082)	Negative	2012 (96.6)	1033 (97.3)	979 (96.0)	0.086
	Positive	70 (3.4)	29 (2.7)	41 (4.0)	
Any higher-risk sex partner in past 6 months^b^	No	1966 (90.2)	945 (75.7)	1004 (94.5)	0.051
	Yes	213 (9.8)	271 (24.3)	59 (5.5)	
Any reported condom use in past 6 months	No	1302 (67.5)	809 (72.5)	854 (80.3)	0.001
	Yes	626 (32.5)	307 (27.5)	209 (19.7)	

CT, *Chlamydia trachomatis*; NG, *Neisseria gonorrhoeae*; STI, sexually transmitted infection.

a STI: active CT and/or NG infection at the time of data collection, as determined by the GeneXpert CT/NG© assay using urine samples.

b Casual sex partner, one-night stand, commercial sex worker/client, or inherited partner.

**Table 4:** Associations between age-disparate relationship classifications and sexual health risk behaviors in rural Uganda and Kenya by sex^a^.

Characteristic STI infection (N=860)	Category	aOR (95% CI)	*P*-value	aOR (95% CI)	*P*-value
		**Male (N=860)**		**Female (N=930)**	
Age disparity of reported relationships	Age-disparate (≥5)	1	-	1	-
	Both (disparate and homogeneous)	0.54 (0.28–1.02)	0.057	3.82 (1.46–9.98)	0.006
	Age-homogeneous (0–4)	0.36 (0.09–1.50)	0.161	2.87 (1.18–6.98)	0.020
Concurrent age-disparate relationships in past 6 months vs none	Yes	1.46 (0.61–3.51)	0.397	5.37 (1.91–15.08)	0.001
Concurrent age-homogeneous relationships in past 6 months vs none	Yes	0.81 (0.23–2.82)	0.736	4.64 (2.15–9.98)	<0.001
Concurrent cross-generational relationships in past 6 months vs none	Yes	1.79 (0.65–4.91)	0.258	6.18 (1.54–24.83)	0.010
Mutually exclusive current age-disparity classifications	Nonconcurrent age-homogeneous	1		1	
	Concurrent age-homogeneous	-	-	1.99 (0.23–17.40)	0.533
	Concurrent age-disparate	1.43 (0.16–13.04)	0.749	2.79 (0.64–12.27)	0.174
	Concurrent cross-generational	3.31 (0.46–23.66)	0.233	4.95 (1.35–18.19)	0.016
**Higher-risk relationship**					
		Male (N=1116)		Female (N=1063)	
Age disparity of reported relationships	Age-disparate (≥5)	1	-	1	-
	Both (disparate and homogeneous)	1.57 (1.00–2.48)	0.052	1.24 (0.75–2.03)	0.406
	Age-homogeneous (0–4)	0.85 (0.51–1.40)	0.529	1.39 (0.72–2.67)	0.322
Concurrent age-disparate relationships in past 6 months vs none	Yes	18.51 (10.68–32.07)	<0.001	13.19 (4.96–35.05)	<0.001
Concurrent age-homogeneous relationships in past 6 months vs none	Yes	7.64 (4.74–12.32)	<0.001	15.35 (7.17–32.85)	<0.001
Concurrent cross-generational relationships in past 6 months vs none	Yes	14.90 (10.16–21.84)	<0.001	15.10 (6.64–34.34)	<0.001
Mutually exclusive concurrent age-disparity classifications	Nonconcurrent age-homogeneous	1		1	
	Concurrent age-homogeneous	19.25 (3.12–118.74)	0.001	64.95 (14.06–300.05)	<0.001
	Concurrent age-disparate	13.02 (5.06–33.46)	<0.001	8.42 (1.04–68.23)	0.046
	Concurrent cross-generational	17.77 (7.00–45.19)	<0.001	16.55 (5.99–45.70)	<0.001
Any condom use in the last 6 months					
		Male (N=1033)		Female (N=895)	
Age disparity of reported relationships	Age-disparate (≥5)	1	-	1	-
	Both (disparate and homogeneous)	1.04 (0.67–1.62)	0.854	1.27 (1.00–1.61)	0.054
	Age-homogeneous (0–4)	0.69 (0.47–1.00)	0.050	1.55 (1.03–2.33)	0.035
Concurrent age-disparate relationships in past 6 months vs none	Yes	4.02 (2.54–6.37)	<0.001	2.57 (0.87–7.55)	0.086
Concurrent age-homogeneous relationships in past 6 months vs None	Yes	2.85 (1.89–4.31)	<0.001	2.99 (1.52–5.89)	0.002
Concurrent cross-generational relationships in past 6 months vs none	Yes	3.67 (2.50–5.39)	<0.001	3.23 (1.16–9.04)	0.025
Mutually exclusive current age-disparity classifications	Nonconcurrent age-homogeneous	1	-	1	-
	Concurrent age-homogeneous	2.37 (0.54–10.38)	0.252	12.26 (1.90–79.07)	0.008
	Concurrent age-disparate	4.69 (2.44–9.00)	<0.001	1.32 (0.18–9.58)	0.785
	Concurrent cross-generational	4.21 (2.25–7.86)	<0.001	2.72 (0.95–7.84)	0.063

aOR, adjusted odds ratio; CI, confidence interval; STI, sexually transmitted infection.

a Individual models for the listed independent variable are adjusted for age, occupation, wealth index, HIV status, any mobility in the past 6 months, region, and community clustering.
